# Recommendations on Complementary Food Introduction Among Pediatric Practitioners

**DOI:** 10.1001/jamanetworkopen.2020.13070

**Published:** 2020-08-17

**Authors:** Waheeda Samady, Emily Campbell, Ozge Nur Aktas, Jialing Jiang, Alexandria Bozen, Jamie L. Fierstein, Alanna Higgins Joyce, Ruchi S. Gupta

**Affiliations:** 1 Ann and Robert H. Lurie Children’s Hospital, Chicago, Illinois; 2 Center for Food Allergy & Asthma Research, Feinberg School of Medicine, Northwestern University, Chicago, Illinois; 3 Department of Pediatrics, Feinberg School of Medicine, Northwestern University, Chicago, Illinois; 4 Department of Pediatrics, University of Illinois College of Medicine at Chicago, Chicago, Illinois; 5 Department of Medicine, Feinberg School of Medicine, Northwestern University, Chicago, Illinois

## Abstract

**Importance:**

The American Academy of Pediatrics and the Centers for Disease Control and Prevention recommend waiting 3 to 5 days between the introduction of new complementary foods (solid foods introduced to infants <12 months of age), yet with advances in the understanding of infant food diversity, the guidance that pediatric practitioners are providing to parents is unclear.

**Objective:**

To characterize pediatric practitioner recommendations regarding complementary food introduction and waiting periods between introducing new foods.

**Design, Setting, and Participants:**

In this survey study, a 23-item electronic survey on complementary food introduction among infants was administered to pediatric health care professionals from February 1 to April 30, 2019. Responses were described among the total sample and compared among subgroups. Survey invitations were emailed to 2215 members of the Illinois Chapter of the American Academy of Pediatrics and the national American Academy of Pediatrics’ Council on Early Childhood. Participants were required to be primary medical practitioners, such as physicians, resident physicians, or nurse practitioners, providing pediatric care to infants 12 months or younger.

**Main Outcomes and Measures:**

The main outcome measures were recommendations on age of complementary food introduction and waiting periods between the introduction of new foods. Categorical survey items were reported as numbers (percentages) and 95% CIs. Means (SDs) were used to describe continuous survey items.

**Results:**

The survey was sent to 2215 practitioners and completed by 604 (response rate, 27.3%). Of these respondents, 41 were excluded because they did not provide care for infants or pediatric patients. The final analyses included responses from 563 surveys. Of these, 454 pediatricians (80.6%), 85 resident physicians (15.1%), and 20 nurse practitioners (3.6%) completed the survey. Only 217 practitioners (38.6%; 95% CI, 34.1%-44.6%) recommended waiting 3 days or longer between food introduction; 259 practitioners (66.3%; 95% CI, 61.4%-70.8%) recommended waiting that amount of time for infants at risk for food allergy development (*P* = .02). A total of 264 practitioners (46.9%; 95% CI, 42.8%-51.0%) recommended infant cereal as the first food, and 226 practitioners (40.1%; 95% CI, 36.1%-44.2%) did not recommend a specific order. A total of 268 practitioners (47.6%; 95% CI, 43.5%-51.7%) recommended food introduction at 6 months for exclusively breastfed (EBF) infants, and 193 (34.3%; 95% CI, 30.5%-38.3%) recommended food introduction at 6 months for non-EBF infants (*P* < .001); 179 practitioners (31.8%; 95% CI, 28.1%-35.8%) recommended food introduction at 4 months for EBF infants, and 239 practitioners (42.5%; 95% CI, 38.4%-46.6%) recommended food introduction at 4 months for non-EBF infants (*P* < .001). A need for additional training on complementary food introduction was reported by 310 practitioners (55.1%; 95% CI, 50.9%-59.1%).

**Conclusions and Relevance:**

In this survey study, most pediatric practitioners did not counsel families to wait 3 days or longer between introducing foods unless infants were at risk for food allergy development. The findings suggest that the current recommendation limits infant food diversity and may delay early peanut introduction. Because the approach to food allergy prevention has changed, a reevaluation of published feeding guidelines may be necessary.

## Key Points

**Question:**

Is it appropriate for pediatric practitioners to recommend waiting several days between the introduction of new foods considering the current emphasis on incorporating a wide variety of foods during infancy to prevent food allergy development?

**Findings:**

In this survey study of 563 pediatric practitioners, 217 (39%) recommended waiting 3 days or longer before introducing new foods; however, for infants at risk for developing food allergy, 259 (66%) recommended waiting. Although 264 practitioners (47%) recommended that cereal be introduced first, 226 (40%) did not recommend any specific order during food introduction.

**Meaning:**

This study found that there was variability among pediatric practitioners’ recommendations on infant diet, suggesting that a reevaluation of published guidelines may be warranted.

## Teaser

This survey study characterizes pediatric practitioner recommendations regarding complementary food introduction and waiting periods between the introduction of new foods.

## Introduction

Introduction of complementary foods (ie, solid foods introduced to infants between the ages of 4 and 11 months to complement breastfeeding and/or formula feeds) is a fundamental discussion every pediatrician has with families of young infants. The American Academy of Pediatrics (AAP) recommends introduction of solid foods between the ages of 4 and 6 months.^1^ The AAP and the Centers for Disease Control and Prevention (CDC) further recommend introducing 1 single-ingredient food at a time and observing the infant for 3 to 5 days between the introduction of each new food to monitor for allergic reactions.^1,2^ Although it is important to monitor for adverse food reactions in infants, it is unclear why specifically a 3- to 5-day period is recommended.

The literature^3,4,5,6,7,8,9,10^ suggests that a diverse diet in the first year of life and early introduction of certain allergenic foods is beneficial to an infant and is associated with a reduced risk of atopy. The landmark Learning Early About Peanut Allergy study^11^ has reinforced the importance of complementary food introduction in early infancy, finding an 86% relative risk reduction in peanut allergy development if peanut-containing foods were introduced between 4 and 11 months of age. Consequently, the National Institute of Allergy and Infectious Diseases (NIAID) published guidelines recommending peanut allergy risk assessment and early introduction of infant-safe peanut products between 4 and 6 months of age for high-risk infants.^12^ However, the current published feeding recommendations may hinder the rapid introduction of a diverse diet and negatively affect the timely coordination of early peanut introduction in infants.

Prior studies^13,14,15,16,17^ regarding infant feeding have focused on parental preference and practices on the timing of complementary food introduction. Several studies^14,17^ report that parents value both family and peer opinions, as well as physician recommendations regarding solid food introduction. However, the manner in which pediatricians recommend introducing complementary food in daily practice has, to our knowledge, not yet been evaluated. The objective of this study was to describe current pediatric practitioners’ recommendations regarding complementary food introduction, focusing on food type, age of introduction, and waiting periods between the introduction of new foods.

## Methods

This survey study was performed from February 1 to April 30, 2019. An electronic survey on solid food introduction among infants was administered to members of the Illinois Chapter of the American Academy of Pediatrics (ICAAP) and the national AAP’s Council on Early Childhood (COEC). The study was reviewed and approved by the institutional review board of the Ann and Robert H. Lurie Children’s Hospital of Chicago and was determined to be exempt because it was not human subjects research. Consent was implied by survey completion, and all data were deidentified. This study followed the American Association for Public Opinion Research (AAPOR) reporting guideline.

### Survey Development

The survey was designed by a team of pediatricians and expert pediatric health service researchers. The instrument was pretested via interviews with pediatricians practicing in the Chicago area. A separate internal research team then iteratively reviewed and revised the instrument in accordance with the pediatricians’ feedback regarding the survey’s readability, layout, length, and appropriateness. The final survey instrument contained 25 items (eAppendix in the Supplement) that evaluated recommendations regarding food introduction type, timing, and waiting periods; sources of guidance that pediatricians used to develop their recommendations; their experience with food allergies; and how their recommendations would change if food allergy risk factors were present.

### Survey Distribution

Surveys were electronically administered via email using REDCap (Research Electronic Data Capture) to members of the ICAAP (n = 1750) and COEC (n = 465). Participants were required to be nonretired primary medical practitioners, such as pediatric or family medicine physicians, resident physicians, or nurse practitioners, providing pediatric care to infants 12 months or younger. Information sheets that described the study’s objectives and participation requirements were shared online before participation in the survey. Participants were offered a $5 gift card for completing the survey. Emails were sent out twice, and all survey responses were anonymous.

### Measures

The primary variables of interest were pediatric practitioners’ current recommendations on complementary food type, age of introduction, and waiting periods (eAppendix in the Supplement). Specifically, age of introduction was based on survey items that asked at approximately what age solid food was recommended for exclusively breastfed (EBF) infants and non-EBF infants. Response categories included ages of 4, 5, 6, and 7 months and other; these categories were chosen to coincide with the AAP recommendation to introduce foods when infants are developmentally ready (approximately 4-6 months of age). For EBF infants, the AAP recommends waiting until 6 months of age to introduce complementary foods.

In addition, respondents’ demographic information was collected; other variables of interest included modification of current recommendations according to infant food allergy risk, personal beliefs about food introduction guidelines on complementary food introduction, and assessment of the need for nutrition training among the medical community.

### Statistical Analysis

The primary objective of this study was to describe current recommendations regarding complementary food introduction among a sample of pediatric practitioners, with a focus on food type, timing, and waiting periods. Categorical survey items were reported as numbers (percentages) and 95% CIs. Means (SDs) were used to describe continuous survey items. With respect to survey items with missing data, numbers and/or means were calculated using the denominator of those who answered the particular survey item. One-sample tests were used to determine differences in proportions between survey item responses. Data from this survey were not weighted.

In addition to the primary analyses, we explored current recommendations preferred among sample subgroups. We used binary variables (yes or no) to assess whether practitioners had graduated medical school within 10 years, had an academic affiliation, and did or did not wait more than 2 days between introducing new foods. We used χ^2^ tests to evaluate unadjusted associations between categorical survey items and binary indicators of each subgroup. A 2-sided *P* < .05 was considered to be statistically significant. The statistical analyses were performed using Stata SE, version 15.1 (StataCorp LLC).

## Results

### Characteristics of Survey Participants

The survey was sent to 2215 practitioners and completed by 604 (response rate, 27.3%). Of these respondents, 41 were excluded because they did not provide care for infants or pediatric patients. The final analyses included responses from 563 surveys. Respondents were mainly pediatricians (454 [80.6%; 95% CI, 77.2%-83.7%]), followed by resident physicians (85 [15.1%; 95% CI, 12.4%-18.3%]), nurse practitioners (20 [3.6%; 95% CI, 2.3%-5.4%]), and family medicine practitioners (4 [0.7% [95% CI, 0.3%-1.9%]). Participants were primarily from the Midwest (446 [81.7%; 95% CI, 78.2%-84.7%]), and 334 (61.3%; 95% CI, 57.1%-65.3%) reported that they had graduated medical school more than 10 years before (Table 1). Practice type varied among participants, with substantial numbers providing care at academic institutions (167 [29.8%; 95% CI, 26.1%-33.7%]) and hospital-affiliated clinics (96 [17.1%; 95% CI, 14.2%-20.5%]). A total of 207 practitioners (37.0%; 95% CI, 32.9%-40.8%) reported that most of their patient population (>50%) was covered by Medicaid. Compared with a 2016 national report of AAP members (n = approximately 67 000),^18^ our surveyed population had a similar number of practitioners in the various practice types (AAP-reported private: 33.3% vs 213 [38.0%], AAP-reported hospital affiliated: 14.6% vs 96 [17.1%], and AAP-reported community health: 3.1% vs 48 [8.6%]), with the exception of our survey population having a higher number of practitioners in academic settings (AAP-reported 15.1% vs 167 [29.8%]) and a lower number in private solo practice (10.5% vs 18 [3.2%]). Hours spent per week working in pediatric care were similar between the surveyed population and AAP members (mean [SD], 36.6 [17.5] vs 32 hours).^18^

**Table 1.** Demographic Data

Variable	Pediatric practitioners,^a^ No. (%) [95% CI]			
	All		Not following guideline to wait ≥3 d between new food introduction	
Practice type (n = 561/224)^b^				
Private group	213 (38.0) [34.0-42.1]		78 (34.8) [28.9-41.3]	
Academic	167 (29.8) [26.1-33.7]		73 (32.6) [26.7-39.0]	
Hospital-affiliated clinic	96 (17.1) [14.2-20.5]		39 (17.4) [13.0-23.0]	
Community health	48 (8.6) [6.5-11.2]		22 (9.8) [6.5-14.5]	
Private solo	18 (3.2) [2.0-5.0]		3 (1.3) [0.4-4.0]	
Managed care or HMO	9 (1.6) [0.8-3.1]		5 (2.2) [0.9-5.3]	
Other	10 (1.8) (1.0-3.3)		4 (1.8) [0.6-4.7]	
Medical specialty (n = 563/224)^b^			
Pediatrics	454 (80.6) [77.2-83.7]		188 (83.9) [78.1-87.9]	
Resident physicians	85 (15.1) [12.4-18.3]		30 (13.4) [9.8-19.0]	
Nurse practitioners	20 (3.6) [2.3-5.4]		4 (1.8) [0.6-4.7]	
Family medicine	4 (0.7) [0.3-1.9]		2 (0.9) [0.2-3.5]	
Year of graduation (n = 545/221)^b^				
2009-2019	211 (38.7) [34.7-42.9]		81 (36.7) [30.5-43.2]	
Earlier than 2009	334 (61.3) [57.1-65.3]		140 (63.3) [56.8-69.5]	
Medicaid patients, % (n = 560/223)^b^				
0-25	238 (42.5) [38.5-46.6]		91 (40.8) [34.5-47.4]	
26-50	87 (15.5) [12.8-18.8]		42 (18.8) [14.2-24.5]	
51-75	88 (15.7) [12.9-19.0]		33 (14.8) [10.7-20.1]	
76-100	119 (21.3) [18.1-24.8]		45 (20.2) [15.4-26.0]	
Do not know	28 (5.0) [3.5-7.2]		12 (5.4) [3.1-9.3]	
WIC patients, % (n = 559/222)^b^				
0-25	251 (44.9) [40.8-49.1]		99 (44.6) [38.2-51.2]	
26-50	75 (13.4) [10.8-16.5]		32 (14.4) [10.4-19.7]	
51-75	78 (14.0) [11.3-17.1]		30 (13.5) [9.5-18.7]	
76-100	97 (17.4) [14.4-20.7]		37 (16.7) [12.3-22.2]	
Do not know	58 (10.4) [8.1-13.2]		24 (10.8) [7.3-15.7]	
Region (n = 546/224)^b^	(n = 546)		(n = 217)	
Midwest	446 (81.7) [78.2-84.7]		173 (79.7) [73.8-84.6]	
Northeast	46 (8.4) [6.4-11.1]		13 (6.0) [3.5-10.1]	
West	41 (7.5) [5.6-10.0]		25 (11.5) [7.9-16.5]	
South	13 (2.4) [1.4-4.1]		6 (2.8) [1.2-6.0]	
Time spent on pediatric care, mean (SD), h/wk	36.6 (17.5)		35.5 (17.1)	

Abbreviations: HMO, health maintenance organization; WIC, Special Supplemental Nutrition Program for Women, Infants, and Children.

^a^ Data are presented as number (percentage) [95% CI] unless otherwise indicated.

^b^ Denominator for each proportion calculation is the number of nonmissing observations denoted in column sample size. n is sample size for all practitioners/sample size for those not following the guideline.

### Solid Food Recommendations

#### Food Type

A total of 264 pediatric practitioners (46.9%; 95% CI, 42.8%-51.0%) recommended infant cereal as the first food of introduction (Table 2). However, 226 practitioners (40.1%; 95% CI, 36.1%-44.2%) reported no recommendation of a specific order. In regard to age at which food should be introduced in EBF and non-EBF infants, 268 practitioners (47.6%; 95% CI, 43.5%-51.7%) recommended 6 months for EBF infants and 193 practitioners (34.3%; 95% CI, 30.5%-38.3%) recommended 6 months for non-EBF infants (*P* < .001); 101 practitioners (17.9%; 95% CI, 15.0%-21.3%) recommended 5 months for EBF infants and 114 practitioners (20.2%; 95% CI, 17.1%-23.8%) recommended 5 months for non-EBF infants (*P* = .20); and 179 practitioners (31.8%; 95% CI, 28.1%-35.8%) recommended 4 months for EBF infants and 239 practitioners (42.5%; 95% CI, 38.4%-46.6%) recommended 4 months for non-EBF infants (*P* < .001).

**Table 2.** Pediatric Solid Food Introduction Recommendations

Variable	Pediatric practitioners, No. (%) [95% CI]				Academic practice, No. (%) [95% CI]		
	All (N = 563)	Graduated >10 y ago (n = 334)	Graduated ≤10 y ago (n = 211)	*P* value^a^	Yes (n = 339)	No (n = 216)	*P* value^a^
First food recommended for introduction							
No recommendation	226 (40.1) [36.1-44.2]	129 (38.6) [34.1-44.6]	91 (43.1) [36.6-50.0]		138 (40.7) [35.6-46.0]	86 (39.8) [33.5-46.5]	
Infant cereal	264 (46.9) [42.8-51.0]	170 (50.9) [45.5-56.2]	84 (39.8) [33.4-46.6]	.01	154 (45.4) [40.2-50.8]	104 (48.2) [41.5-54.8]	.60
Fruits	8 (1.4) [0.7-2.8]	2 (0.6) [0.1-2.4]	6 (2.8) [1.3-6.2]		6 (1.8) [0.8-3.9]	2 (0.9) [0.2-3.7]	
Vegetables	45 (8.0) [6.0-10.5]	20 (6.0) [3.9-9.1]	24 (11.4) [7.7-16.4]		28 (8.3) [5.8-11.7]	17 (7.9) [4.9-12.3]	
Meats	4 (0.7) [0.3-1.9]	2 (0.6) [0.1-2.4]	2 (1.0) [0.2-3.7]		4 (1.2) [0.4-3.1]	0 (-)	
Other	16 (2.8) [1.0-3.3]	11 (3.3) [1.8-5.9]	4 (1.9) [0.1-4.9]		9 (2.7) [1.4-5.0]	7 (3.2) [1.5-6.7]	
Timing of introduction for exclusively breastfed infants, mo							
4	179 (31.8) [28.1-35.8]	98 (29.3) [24.7-34.5]	74 (35.1) [28.9-41.8]	.20	106 (31.3) [26.5-36.4]	70 (32.4) [26.5-38.9]	.80
5	101 (17.9) [15.0-21.3]	66 (19.8) [15.8-24.4]	33 (15.6) [11.3-21.2]		65 (19.2) [15.3-23.7]	34 (15.7) [11.4-21.3]	
6	268 (47.6) [43.5-51.7]	158 (47.3) [41.9-52.7]	101 (47.9) [41.2-54.6]		159 (46.9) [41.6-52.2]	106 (49.1) [42.4-55.8]	
Other	15 (2.7) [1.6-4.4]	12 (3.6) [2.0-6.2]	3 (1.4) [0.5-4.3]		9 (2.7) [1.4-5.0]	6 (2.8) [1.2-6.1]	
Timing of introduction for nonexclusively breastfed infants, mo							
4	239 (42.5) [38.4-46.6]	133 (39.8) [34.7-45.2]	95 (45.0) [38.4-51.8]	.20	142 (41.9) [36.7-47.2]	93 (43.1) [36.6-49.7]	.60
5	114 (20.2) [17.1-23.8]	72 (21.6) [17.5-26.3]	39 (18.5) [13.8-24.3]		72 (21.2) [17.2-25.9]	41 (18.9) [14.2-24.8]	
6	193 (34.3) [30.5-38.3]	115 (34.4) [29.5-39.7]	74 (35.1) [28.9-41.8]		117 (34.5) [29.6-39.7]	73 (33.8) [27.8-40.4]	
Other	17 (3.0) [1.9-4.8]	14 (4.2) [2.5-7.0]	3 (1.4) [0.5-4.3]		8 (2.4) [1.2-4.7]	9 (4.2) [2.2-7.8]	
Source of guidance							
Training	326 (57.9) [53.8-61.9]	152 (45.5) [40.2-50.9]	167 (79.2) [73.1-84.1]	<.001	206 (60.8) [55.4-65.8]	114 (52.8) [46.1-59.4]	.06
AAP	279 (49.6) [45.4-53.7]	169 (50.6) [45.2 - 55.9]	101 (47.9) [41.2-54.6]	.50	161 (47.5) [42.2-52.8]	114 (52.8) [46.1-59.4]	.20
Professional experience	246 (43.7) [39.6-47.8]	185 (55.4) [49.9-60.7]	53 (25.1) [19.7-31.4]	<.001	136 (40.1) [35.0-45.4]	108 (50.0) [43.3-56.7]	.02
Personal experience	199 (35.4) [31.5-39.4]	140 (41.9) [36.7-47.3]	57 (27.0) [21.4-33.4]	<.001	120 (35.4) [30.5-40.7]	77 (35.6) [29.5-42.3]	.90
Colleagues	129 (22.9) [19.6-26.6]	55 (16.5) [12.9-20.9]	70 (33.2) [27.1-39.8]	<.001	89 (26.3) [21.8-31.2]	39 (18.1) [13.5-23.8]	.03
Medical meetings	85 (15.1) [12.4-18.3]	13 (3.9) [2.3-6.6]	4 (1.9) [0.7-4.9]	.10	8 (2.4) [1.2-4.7]	9 (4.2) [2.2-7.8]	.20
Cultural	63 (11.2) [8.8-14.0]	38 (11.4) [8.4-15.3]	23 (10.9) [7.3-15.9]	.90	44 (13.0) [9.8-17.0]	18 (8.3) [5.3-12.9]	.09
Professional organizations	17 (3.0) [1.9-4.8]	51 (15.3) [11.8-19.6]	30 (14.2) [10.0-19.6]	.70	49 (14.5) [11.1-18.6]	36 (16.7) [12.2-22.3]	.50
Other	15 (2.7) [1.6-4.4]	12 (3.6) [2.0-6.2]	3 (1.4) [0.4-4.3]	.10	7 (2.1) [1.0-4.3]	8 (3.7) [1.9-7.3]	.20
Need for training and education on solid food introduction	310 (55.1) [50.9-59.1]	146 (72.0) [65.6-77.7]	152 (43.7) [38.5-49.1]	<.001	197 (58.1) [52.8-63.2]	107 (49.5) [42.9-56.2]	.05

Abbreviation: AAP, American Academy of Pediatrics.

^a^ *P* values are derived from χ^2^ tests that evaluated associations between the categorical row variable and the binary column variable (ie, graduated within 10 years ago, yes or no; academic practice, yes or no).

#### Time Between Introductions

With respect to time elapsed between food introduction, 217 participants (38.6%; 95% CI, 34.1%-44.6%) recommended waiting 3 or more days, whereas 112 (19.9%; 95% CI, 16.8%-23.4%) recommended waiting 2 days, and 154 (27.4%; 95% CI, 23.8%-31.2%) recommended waiting 1 day (Table 3). A total of 56 practitioners (9.9%; 95% CI, 7.7%-12.7%) recommended introducing multiple new foods in 1 day; however, 340 (60.4%; 95% CI, 56.3%-64.3%) reported that they believed the introduction of multiple new foods (that are not common food allergens) together was safe (Table 4). No significant associations were observed between recommending more than 2 days between food introduction and practitioner demographics, including patient Medicaid population.

**Table 3.** Recommendations for Infants With and Without Food Allergy Risk Factors

Recommendation	Pediatric practitioners, No. (%) [95% CI]	
	General recommendations (n = 563)	Recommendation for infants with food allergy risk factors (n = 391)
Introduce multiple foods in 1 d	56 (9.9) [7.7-12.7]	5 (1.3) [0.5-3.0]
Introduce multiple foods in 1 meal	15 (2.7) [1.6-4.4]	1 (0.3) [0-1.8]
Introduce 1 food a day	154 (27.4) [23.8-31.2]	35 (9.0) [6.5-12.2]
Introduce 1 food, wait 2 d, introduce another	112 (19.9) [16.8-23.4]	61 (15.6) [12.3-19.6]
Introduce 1 food, wait 3 d, introduce another	171 (30.4) [26.7-34.3]	163 (41.7) [36.9-46.7]
Introduce 1 food, wait >3 d	46 (8.2) [6.2-10.7]	96 (24.6) [20.5-29.1]
Other	9 (1.6) [0.8-3.0]	30 (7.7) [5.4-10.8]

**Table 4.** Practitioner Beliefs Regarding Solid Food Introduction and Factors that Would Change Recommendations

Variable	Pediatric practitioners, No. (%) [95% CI]			Academic practice, No. (%) [95% CI]				
	All (N = 563)	Graduated >10 y ago (n = 334)	Graduated ≤10 y ago (n = 211)		*P* value^a^	Yes (n = 339)	No (n = 216)	*P* value^a^
Think it is safe to introduce multiple foods (nontop allergens) together	340 (60.4) [56.3-64.3]	208 (62.3) [56.9-67.3]	124 (58.8) [52.0-65.3]		.40	212 (62.5) [57.2-67.5]	123 (56.9) [50.2-63.4]	.20
Think waiting between basic food introduction (nontop allergens) is helpful for families	313 (55.6) [51.4-59.7]	181 (54.2) [48.8-59.9]	121 (57.4) [50.5-63.9]		.20	178 (52.5) [47.2-57.8]	132 (61.1) [54.4-67.4]	.10
Factors that would change recommendation								
Older sibling with food allergy	387 (68.7) [64.8-72.4]	226 (67.7) [62.4-72.5]	149 (70.6) [64.1-76.4]		.50	237 (69.9) [64.8-74.6]	146 (67.6) [61.0-73.5]	.60
Infant with moderate to severe eczema	374 (66.4) [62.4-70.2]	227 (68.0) [62.7-72.8]	138 (65.4) [58.7-71.5]		.50	236 (69.6) [64.5-74.3]	136 (63.0) [56.3-69.2]	.10
Family history of food allergy	370 (65.7) [61.7-69.5]	213 (63.8) [58.5-68.8]	146 (69.2) [62.6-75.1]		.20	228 (67.3) [62.1-72.1]	136 (63.0) [56.3-69.2]	.30
Family history of any allergies or asthma	165 (29.3) [25.6-33.2]	89 (26.7) [22.2-31.7]	71 (33.7) [27.6-40.3]		.08	105 (31.0) [26.3-36.1]	58 (26.9) [21.3-33.2]	.30
Child has any eczema	86 (15.3) [12.5-18.5]	50 (15.0) [11.5-19.2]	35 (16.6) [12.1-22.3]		.60	50 (14.8) [11.3-19.0]	34 (15.7) [11.4-21.3]	.70
Other	20 (3.6) [2.3-9.8]	13 (3.9) [2.3-6.6]	5 (2.4) [1.0-5.6]		.30	12 (3.5) [2.0-6.1]	8 (3.7) [1.9-7.3]	.90
Infants with food allergy in past year, %								
0	113 (20.1) [17.0-23.6]	55 (16.5) [12.9-20.9]	54 (25.6) [20.1-31.9]		.20	82 (24.2) [19.9-29.1]	28 (13.0) [9.1-18.2]	.02
<5	314 (55.8) [51.6-59.8]	194 (58.1) [52.7-63.3]	110 (52.1) [45.4-58.8]			170 (50.2) [44.8-55.5]	141 (65.3) [58.7-71.4]	
5-10	110 (19.5) [16.4-23.0]	67 (20.1) [16.0-24.7]	40 (19.0) [14.2-24.9]			72 (21.2) [17.2-25.9]	36 (16.7) [12.2-22.3]	
11-20	20 (3.6) [2.3-5.4]	15 (4.5) [2.7-7.3]	5 (2.4) [1.0-5.6]			11 (3.2) [1.8-5.8]	9 (4.2) [2.2-7.8]	
21-40	2 (0.4) [0.1-1.4]	1 (0.3) [0-2.1]	1 (0.5) [0.1-3.3]			0 (-)	2 (0.9) [0.2-3.7]	
>40	1 (0.2) [0-1.3]	1 (0.3) [0-2.1]	0 (-)			1 (0.3 [0.04-2.1]	0 (-)	
Other	3 (0.5) [0.2-1.6]	1 (0.3) [0-2.2]	1 (0.5) [0.1-3.3]			3 (0.9) [0.3-2.7]	0 (-)	

^a^ *P* values are derived from χ^2^ tests that evaluated associations between the categorical row variable and the binary column variable (ie, graduated within 10 years ago, yes or no; academic practice, yes or no).

### Food Allergy Risk Factors and Solid Food Introduction

Overall, 391 practitioners (69.4%; 95% CI, 65.5%-73.1%) reported they would change their current recommendation because an infant had risk factors for food allergy development, including older siblings with food allergy (387 [68.7%; 95% CI, 64.8%-72.4%]), moderate to severe eczema (374 [66.4%; 95% CI, 62.4%-70.2%]), and family history of food allergy (370 [65.7%; 95% CI, 61.7%-69.5%]) (Table 4). A total of 163 practitioners (41.7%; 95% CI, 36.9%-46.7%) recommended waiting 3 days between food introduction among infants with food allergy risk factors, whereas 96 practitioners (24.6%; 95% CI, 20.5%-29.1%) recommended waiting more than 3 days. A total of 35 practitioners (9.0%; 95% CI, 6.5%-12.2%) recommended waiting 1 day between the introduction of new foods for infants with food allergy risk factors. Practitioners more frequently recommended waiting 3 or more days in children with food allergy risk factors than they did in children without these risks (259 [66.3%] vs 217 [38.6%], *P* = .02). Food-related allergic reactions among infants during complementary food introduction occurred infrequently, with 314 practitioners (55.8%; 95% CI, 51.6%-59.8%) reporting they had occurred in less than 5% of infants and 110 (19.5%; 95% CI, 16.4%-23.0%) reporting they had occurred in 5% to 10% of infants.

### Need for Further Education

Additional training and education on complementary food introduction were identified as a need by 310 participants (55.1%; 95% CI, 50.9%-59.1%) overall and by 146 participants (72.0%; 95% CI, 65.6%-77.7%) who graduated medical school more than 10 years before the survey. Pediatric residency training (326 [57.9%; 95% CI, 53.8%-61.9%]), AAP guidelines (279 [49.6%; 95% CI, 45.4%-53.7%]), and professional experience (246 [43.7%; 95% CI, 39.6%-47.8%]) were the 3 most common reported sources of guidance in developing their complementary food recommendations (Table 2).

## Discussion

To our knowledge, this survey study is the first study to evaluate recommendations on complementary food introduction by pediatric practitioners. We found that most practitioners recommended waiting 2 days or less between the introduction of new foods, with only 2 of 5 reported following the AAP and CDC recommendation of waiting 3 to 5 days between the introduction of new foods. Most also believed that administering multiple nonallergenic foods was safe, although only 2.7% recommended it. Despite reporting that food-related reactions occurred infrequently among infants, most practitioners recommended waiting longer and complying with AAP recommendations for infants with food allergy risk factors, with 66.3% recommending 3 or more days between the introduction of new foods. Common sources of guidance for recommendations reported by practitioners were primarily clinical experience (both medical training and professional experience), and 55.1% reported a need for additional training in this area.

Previous studies^14,17^ of parents regarding feeding practices identified pediatricians as trusted sources of information by parents during the solid food introduction phase; however, data are limited on how pediatric practitioners provide recommendations on complementary foods. Our study findings support those of previous studies^14,16^ that evaluated parental feeding practices. For example, the Infant Feeding Practices Study II,^16^ which analyzed parental feeding practices in a large cohort of infants during the first year of life, found that at 4 months of age, 40% of infants had consumed infant cereal and 17% had consumed fruits and/or vegetables. This finding correlates with our study’s finding that 46.9% of pediatric practitioners recommended infant cereal as the first food and 42.5% recommended initiation of complementary foods at 4 months of age in non-EBF infants. This association between parental practices and physician recommendations suggests that anticipatory guidance with evidence-based practices is of value to parents and their infants.

Our study found that most practitioners diverged from published guidelines in their recommendation to wait 3 to 5 days between the introduction of each new food.^1,2^ We found that many pediatric practitioners recommended waiting 2 days or less before starting a new food. Of note, although 60.4% of practitioners indicated that they believe administering multiple nonallergenic foods is safe, in clinical practice, only 1 in every 10 practitioners reported recommending multiple foods a day, and only 2.7% recommended giving multiple foods in 1 meal. The discrepancy between practitioners’ beliefs and clinical recommendations found in this study may represent the conflict between training and professional experiences and the current guidelines. This discrepancy, in addition to the scarcity of evidence supporting the current recommendations on how frequently new foods can be introduced to infants, reinforces the need to reevaluate the current recommendations.

Pediatric practitioners reported low rates of food-related allergic reactions in our study, a finding that comports with recent population-based estimates of food allergy burden among infants.^19^ Assessment of allergic reactions is an important consideration during solid food introduction. Per the American Academy of Allergy, Asthma, and Immunology, this cautionary waiting period is to evaluate for adverse reactions during introduction of a new food, with a goal of identifying foods that cause an allergic reaction.^20^ However, a 3-day waiting period does not match the clinical time course of most allergic reactions. IgE-mediated food reactions typically occur immediately or within 2 hours of ingestion. Non–IgE-mediated reactions, such as food protein–induced enterocolitis syndrome, can manifest as repetitive vomiting in the 1- to 4-hour period after ingestion of the suspect food, in the absence of classic IgE-mediated skin or respiratory symptoms, with cow’s milk and soy being the most common triggers.^21,22,23,24^ Most other non–IgE-mediated food allergies follow a short-term or long-term timeline but can be managed by elimination of the most common suspected antigens, instead of introducing all foods with significant waiting periods. Furthermore, more than 90% of food allergies are caused by the top 8 allergens (peanut, tree nut, egg, milk, soy, wheat, fish, and shellfish) rather than cereal, fruits, vegetables, and meat, which were found in our study to be introduced first to infants.^19,25^

Our study found that only 55.6% of practitioners believed that the recommendation to wait several days before introducing each new food was helpful to families. In practice, this recommendation may have a deleterious effect of limiting early infant food diversity, which has been reported to be associated with an increased risk of pediatric asthma and allergies.^3,4,6,7,8^ Introducing a more diverse diet in the first year of life has been shown to be associated with a reduced risk of atopic dermatitis, asthma, and food allergies up to 6 years of age.^3,4,8^ Specifically, late introduction of certain foods, including potatoes, oats, rye, wheat, meat, fish, and eggs, has been reported to be directly associated with sensitization to food allergens.^6^ Moreover, early introduction of grains, fish, and egg has been shown to be associated with decreased risks of asthma, allergic rhinitis, and atopic sensitization.^7^ Under the current guidelines, an infant is only exposed to 5 to 7 new foods a month, which can significantly limit infant food exposure. This diversity might be increased if a new food could be introduced daily and would support the current CDC general nutrition recommendation to increase food exposure in the first year and avoid picky eater syndrome.^26^

Reevaluation of the current waiting period should also include consideration of how to incorporate infant feeding of peanut-containing foods into an infant’s diet. A total of 259 surveyed practitioners recommended waiting longer for infants with food allergy risk factors, with 66.3% recommending 3 or more days between the introduction of new foods. In contrast, the NIAID currently recommends infants with atopic dermatitis be given peanut-containing foods between 4 and 6 months of age,^12^ with the intent of exposing them to peanut via their intestinal tract before sensitization via their skin.^27^ Of note, these guidelines specifically state that several new foods should be introduced before peanut. If infants are required to wait 3 to 5 days between the introduction of each new food, peanut introduction may be delayed past the recommended ages.

### Limitations

This study has limitations. Our data are based on self-reported survey responses. Questions that target the source of guidance for complementary food introduction and the prevalence of food allergy seen in the practitioners’ clinical practice might have been subjected to response bias. To minimize response bias, survey participants’ answers were anonymous. Of note, our findings are more likely to be an underestimate than an overestimate of the lack of consistency with AAP recommendations among US pediatricians more broadly because participants were recruited via AAP LISTSERVS. Our survey had a response rate of 27.3%; however, because the survey was distributed to practicing practitioners via email, this response rate is consistent with a similar study.^28^ Although we were unable to compare responders and nonresponders of the specific AAP LISTSERVS in which the survey was distributed, we provide comparisons between responders and national AAP members and found many similarities in practice type and work hours. The survey population had a higher percentage of academic pediatricians than the overall national membership does, which should be noted when interpreting the results of this study. In addition, most responses were from Midwestern practitioners, so the results may not be entirely representative of practitioners nationally. Also, our study was conducted shortly after the release of the 2017 NIAID peanut prevention guidelines; although pediatric practitioners may have been aware of these new recommendations, it is possible that they did not affect the guidance practitioners provided patients on general food introduction. This guidance may change as these guidelines are more widely adopted.

## Conclusions

In this survey study, most pediatric practitioners did not counsel families to wait 3 days or longer between introducing foods unless infants were at risk for food allergy development. The findings suggest that the current recommendation limits infant food diversity and may delay early peanut introduction. Because the approach to food allergy prevention has changed, a reevaluation of published feeding guidelines may be necessary. Further research appears to be needed to explore an introduction schedule that is evidenced based, safe, and practical for infants and their families.

## Notes

Supplement. **eAppendix.** Final Survey Instrument
