# Error in Byline and Affiliations

**DOI:** 10.1001/jamanetworkopen.2023.40509

**Published:** 2023-10-17

In the Original Investigation by Samady et al titled “Recommendations on Complementary Food Introduction Among Pediatric Practitioners,”^1^ published August 17, 2020, the surname and academic degrees of coauthor Alanna Higgins Joyce were incorrectly styled in the byline. They now appear as “Higgins Joyce, MD, MSE.” The academic degrees of coauthor Waheeda Samady, MD, MSci, were also incorrectly styled. These errors caused the coauthors’ affiliation with the Ann and Robert H. Lurie Children’s Hospital in Chicago, Illinois, not to appear. This article has been corrected.^1^
