# Supplementary material for: Recommendations on Complementary Food Introduction Among Pediatric Practitioners
Source: JAMA Netw Open. 2020 Aug 17;3(8):e2013070. doi: 10.1001/jamanetworkopen.2020.13070 (PMC7431991; doi:10.1001/jamanetworkopen.2020.13070)
Supplement: Supplement. — eAppendix. Final Survey Instrument [file jamanetwopen-3-e2013070-s001.pdf]

Supplementary Online Content

Samady W, Campbell E, Aktas ON, et al. Recommendations on complementary food introduction among pediatric practitioners. *JAMA Netw Open*. 2020;3(8):e2013070. doi:10.1001/jamanetworkopen.2020.13070

eAppendix. Final Survey Instrument

This supplementary material has been provided by the authors to give readers additional information about their work.

© 2020 Samady W et al. *JAMA Network Open.*

eAppendix. Final Survey Instrument

Q. What is your primary medical specialty?

a. Pediatrics

b. Allergy and Immunology (End survey, provide message)

c. Family Medicine

d. Internal Medicine (End survey, provide message)

e. Other (End survey, provide message)

(Message: Provide a thank-you message stating that the survey is targeting pediatricians and that he/she will receive the compensation stated in the invitation letter)

Q. Do you provide general pediatric care to infants ≤12 months of age?

a. Yes

b. No (End survey; provide a thank-you message stating that the survey is targeting pediatricians in general practice who provide care to infants and that he/she will receive the compensation stated in the invitation letter)

Q. What is the first food you recommend that parents introduce to their infants?

a. Infant cereal

b. Vegetables

c. Fruits

d. Meats

e. I tell them it doesn't matter what they introduce first

f. Other: (Text box)

Q. At what approximate age do you recommend introduction of solid foods for exclusively breast fed infants who are developmentally ready?

a. 4 months

b. 5 months

c. 6 months

© 2020 Samady W et al. *JAMA Network Open.*

d. 7 months

e. Other (Text box)

Q. At what approximate age do you recommend introduction of solid foods for formula fed (or not exclusively breast fed) infants who are developmentally ready?

a. 4 months

b. 5 months

c. 6 months

d. 7 months

e. Other (Text box)

Q. How do you recommend introducing solid foods to healthy infants who are developmentally ready?

a. Introduce one food, wait one day, introduce another

b. Introduce one food, wait two days, introduce another

c. Introduce one food, wait three days, introduce another

d. Introduce one food, wait more than three days, introduce another

e. Introduce multiple foods in one day

f. Introduce multiple foods in one meal

g. Other: (textbox)

Q. Why do you recommend solid food introduction in this way? Select all that apply

a. AAP recommendations

b. Residency/ Fellowship training

c. Previous practice experience

d. Personal experience

e. Medical colleagues

f. Articles or notices from professional organizations

g. Local, state, national, or international medical meetings

© 2020 Samady W et al. *JAMA Network Open.*

h. Cultural practices of patient population

i. Other: (Textbox)

Q. Do you have recommendations about the order in which specific solid foods are introduced?

a. No

b. Yes

c. No specific order, family preference

Q. In what order do you recommend introduction to solids? Please number in order.

a. Fruits

b. Cereal

c. Vegetables

d. Meats

e. Nuts

g. Other

Q. Do you think it is safe for families to introduce multiple foods together if they are foods that are not top allergens?

a. No

b. Yes

Q. In the past year, how many of your patients have reported experiencing an allergic reaction to a food during the solid food introduction phase?

a. 0%

b. <5%

c. 5%-10%

d. 10%-20%

e. 20%-40%

© 2020 Samady W et al. *JAMA Network Open.*

f. >40%

g. Other: (textbox [6%-500%]

Q. What do YOU personally believe is the ideal time to wait before introducing a new complementary foods?

a. Introduce one food, wait one day, introduce another

b. Introduce one food, wait two days, introduce another

c. Introduce one food, wait three days, introduce another

d. Introduce one food, wait more than three days, introduce another

e. Introduce multiple foods in one day

f. Introduce multiple foods in one meal

g. Other (textbox)

Q. Do you think this recommendation of waiting between introducing basic foods (not top allergens) is helpful for families? (Top allergens include - peanuts, tree nuts, shellfish, fin fish, milk, egg, soy wheat)

a. Yes

b. No

Q. Which of the following factors would change your recommendations or approach to solid food introduction? Mark all that apply:

a. Infant having any eczema

b. Infant only with moderate to severe eczema

c. Family history of any allergies, asthma

d. Family history of food allergy

e. Older sibling history of food allergy

f. Other: (textbox)

Q. Do you provide different recommendations regarding solid food introduction for infants with strong risk factors for food allergies?

© 2020 Samady W et al. *JAMA Network Open.*

a. No

b. Yes

Q. How do you counsel caregivers in regards to time in between the introduction of new solid foods if their infant has strong risk factors for food allergies?

a. Introduce one food, wait one day, introduce another

b. Introduce one food, wait two days, introduce another

c. Introduce one food, wait three days, introduce another

d. Introduce one food, wait more than three days, introduce another

e. Introduce multiple foods in one day

f. Introduce multiple foods in one meal

g. Other (textbox)

Q. Do you believe you need more education or training on solid food introduction to infants?

a. Yes

b. No

Q. In what year did you graduate from medical school?

a. (XXXX)

Q. Are you a resident or fellow in training?

a. Yes

b. No

Q. Is your practice affiliated with a university or other academic institution? (If more than one pediatric practice, the practice at which you spend the most time.)

a. Yes

b. No

© 2020 Samady W et al. *JAMA Network Open.*

Q. What type is your pediatric practice? (If more than one pediatric practice, the practice at which you spend the most time.)

a. Private practice: solo practice

b. Private practice: group practice

c. Academic medical center practice or clinic

d. Hospital practice or clinic (not part of an academic medical center)

e. Community clinic or community health center

f. Managed care organization/HMO

g. Military or U.S. government

h. Other: (textbox)

Q. In which zip code is your practice located?

a. (Text box – numerical)

Q. In a typical week, how many hours do you spend in pediatric patient care?

a. __ Hours per week

Q. Approximately how many of your pediatric patients are on Medicaid (including Medicaid MCOs and traditional Medicaid)?

a. 0-25 %

b. 26- 50%

c. 51-75%

d. 76-100%

e. Unsure/Don't know

Q. How many of your patients qualify for or receive WIC benefits?

a. 0-25 %

© 2020 Samady W et al. *JAMA Network Open.*

b. 26- 50%

c. 51-75%

d. 76-100%

e. Unsure/Don’t know

© 2020 Samady W et al. *JAMA Network Open.*
